# mTOR signaling orchestrates the expression of cytoprotective factors during cellular senescence

**DOI:** 10.18632/oncotarget.10743

**Published:** 2016-07-20

**Authors:** Reut Yosef, Valery Krizhanovsky

**Affiliations:** Department of Molecular Cell Biology, The Weizmann Institute of Science, Rehovot, Israel

**Keywords:** cellular senescence, mTOR, apoptosis, Autophagy

Cellular senescence is an evolutionarily selected physiological mechanism which is necessary during embryonic development and used later in life to limit tissue damage [1, 2]. In the short term, senescence is a protective response that limits tumorigenesis and aids wound healing. However, if senescent cells are not efficiently resolved by the immune system, they accumulate and, in the long term, may cause deleterious pathological consequences by promoting age-related diseases and cancer. In a recent study we demonstrated that senescent cells, induced to senesce via replicative exhaustion, oncogenic insult or DNA damage, upregulate proteins of the anti-apoptotic BCL-2 family in order to resist apoptosis [3]. Combined inhibition of these proteins using siRNAs or a small molecule ABT-737 induced selective death of senescent cells via apoptosis. The senolytic activity of ABT-737 molecule was demonstrated in two independent models of senescence *in vivo*. Importantly, clearance of senescent cells by ABT-737, as well as by its homolog compound ABT-263 in a different setting, resulted in increased proliferation of stem cells [3, 4]. This surprising effect of elimination of senescent cells might lead to improved tissue renewal and tissue fitness and thus contribute to lifespan extension.

Inhibition of the BCL-2 family members can be achieved by a single inhibitor leading to apoptosis of senescent cells. However, the regulation of the expression of these proteins in senescent cells induced by different stimuli is complex. The mammalian target of rapamycin (mTOR) signaling might play an important role in this regulation. This pathway is suppressed in DNA- damaged induced senescent (DIS) cells, while it is highly activated in oncogene-induced senescent (OIS) cells, where it drives the production of many components of senescence-associated secretory phenotype (SASP) [3, 5-7]. Inhibition of mTOR signaling pathway disrupts protein translation by inactivation of ribosomal S6 kinases (S6K1 and S6K2) and by activation of the binding protein of eukaryotic translation initiation factor 4E (4E-BP), thereby preventing 4E-mediated translation of mRNA species possessing a 5′ ‘cap. How then is the expression of the BCL-2 proteins in DIS cells increased under mTOR inhibition? In DIS cells the increase in BCL-XL protein level is achieved by a specific enhanced translation via an internal ribosome entry site (IRES) motif [3]. The increase in BCL-W protein level might be achieved by an increase in its transcription rate. DIS cells upregulate only the transcription of the short variant of BCL-W gene (variant-2), which is transcribed from an alternative transcription start site. Apparently, the 5′-UTR of this isoform is missing a 5′ terminal oligopyrimidine tract (5′TOP) sequence element that is present only on the long and more abundant isoform of the gene (variant-1). The translation of 5′-TOP mRNAs was suggested to be positively regulated by mTOR pathway, possibly putting a selective pressure for translation of BCL-W from its short isoform under mTOR inhibition during DIS and from its long isoform during OIS when mTOR is activated.

The activation of mTOR pathway is considered a hallmark of organismal aging [5]. mTOR inhibition extends life span and improves age-related pathologies in multiple species. Reduction in mRNA translation that allows better maintenance of protein homeostasis is one of the processes contributing to the pro-longevity effects of mTOR inhibition. Importantly, inhibition of mTOR signaling has been linked to enhanced stress resistance, anti-inflammatory effects and rejuvenation of stem-cell function in a variety of tissues. The effects of mTOR inhibition resemble the effect obtained by specific elimination of DIS cells, linking mTOR inhibition and elimination of senescent cells. Future studies will determine if elimination of senescent cells can execute anti-aging effects through mechanisms affected by mTOR inhibition.
